# Vil Du?! incorporation of a serious game in therapy for sexually abused children and adolescents

**DOI:** 10.1186/s13034-021-00377-3

**Published:** 2021-05-25

**Authors:** Joyce J. Endendijk, Henny K. Tichelaar, Menno Deen, Maja Deković

**Affiliations:** 1 Child and Adolescent Studies, Utrecht University, Heidelberglaan 1, 3548 CS Utrecht, The Netherlands; 2 Lapp, Hoveniersstraat 11, 3513 XS Utrecht, The Netherlands

**Keywords:** Child sexual abuse, Psychotherapy, Serious games, Evaluation, Mixed-methods triangulation design

## Abstract

**Background:**

Talking about experiences of sexual abuse in therapy is difficult for children and adolescents, amongst others due to a lack of vocabulary to describe the situation, avoidance, or feelings of shame, fear, and self-blame. The serious game Vil Du?! was developed to help children open up about sexual experiences. Vil Du?! is a non-verbal communication game, which resembles a dress-up game, in which children can show the therapist what happened to them. The current study examined how and for which therapy components Vil Du?! was used by therapists.

**Methods:**

We used a mixed-methods triangulation design. Therapists filled out online surveys about the use of Vil Du?! with 23 clients (*M*_age_ = 11.38 years, *SD* = 3.96; 61 % female). We also conducted semi-structured interviews with 10 therapists. The data were analyzed in SPSS (quantitative) and Nvivo (qualitative) following the stepwise guidelines of Zhang and Wildemuth.

**Results:**

Merged qualitative and quantitative data revealed that therapists acknowledged the usefulness of Vil Du?! mostly for the therapy components trauma narration and processing, and psycho-education about sexuality. In addition, Vil Du?! might be most useful for clients who have difficulty with disclosing sexual abuse experiences, due to limited verbal abilities or feelings of guilt, shame, avoidance, and tension.

**Conclusions:**

Recommendations from this study were incorporated in a user manual as a first step toward more systematic and broad implementation of Vil Du?! in the treatment of young sexual abuse victims. A next step is to test whether implementing Vil Du?! in therapy is effective in reducing the negative mental health consequences of sexual abuse for children and adolescents.

## Background

Worldwide one in eight children (12.7 %; 18 % of girls; 8 % of boys) become victims of sexual abuse each year [1]. Child sexual abuse (CSA) entails any sexual act (i.e., done for physical or mental sexual gratification of the perpetrator, or experienced by the victim as sexual), inflicted to a child (i.e., below the legal age of adulthood, or developmentally a child), for which full, free, voluntary, and unforced consent was not given (i.e., due to lack of ability, or no true consent), and that occurs in the context of a relationship of power, a position of inequality, or exploitation of vulnerability [2]. Experiencing sexual abuse in childhood or adolescence has drastic and long-term consequences, ranging from a higher risk of a range of psychiatric disorders, drug abuse, relationship problems, sexual difficulties, health problems, later abuse of one’s own child [3], and increased risk of premature mortality in middle adulthood [4]. Symptoms can occur directly following the abuse, but victims can also remain asymptomatic (estimated up to 40 %) [5]. The most common disorder in young victims of sexual abuse is post-traumatic stress disorder (PTSD) [6], and child victims referred for therapy experienced high levels of PTSD symptoms [7]. In addition, symptomatology often uniquely related to CSA is sexualized behavior, such as sexual preoccupation or boundary problems [3].

However, early treatment of victims of child sexual abuse experiencing mental health symptoms appears to be effective in reducing the negative consequences of CSA [8, 9]. Therapies including cognitive-behavioral components (CBT) are the most often used and recommended forms of psychotherapy for CSA victims experiencing psychiatric symptoms [10–13]. Yet, CBT relies heavily on abstract thinking about one’s cognitions and detailed verbal narration of one’s experiences. These tasks might be particularly difficult for children who have limited verbal capacities or a lack of vocabulary to describe the sexual and intimate acts they were part of [8].

To increase applicability of CBT with children and adolescents, researchers suggested the use of content that is tailored to the developmental needs of young clients and to emphasize elements of play [14, 15]. Originating from these ideas, serious games (i.e., interactive media that is developed for other purposes than entertainment) have increasingly been incorporated in the context of psychotherapy in the last 10 years [16]. A systematic review concluded that serious games are effective both as a stand-alone intervention or as a tool used in addition to psychotherapy for a wide range of disorders [16].

However, there appear to be no serious games yet that could be used specifically in the treatment of young CSA victims. Therefore, the aim of this study was to conduct an exploratory evaluation among therapists of a newly developed serious game, called Vil Du?! [17], that helps children open up about sexual abuse experiences.

### Use of serious games in psychotherapy for children

The last decade research, as well as clinical practice, has shown an increasing interest in the use of serious games in psychotherapy for adults and children [16, 18]. This interest is not surprising considering that serious games are highly congruent with children’s experience and involvement with the digital world [19]. In addition, the use of games in psychotherapy fits with recommendations that psychotherapy for children should provide an engaging, client-centered experience [20]. Researchers have suggested that serious games “can be used as a ‘third party in the room’, helping to make the therapeutic process less difficult for adolescents by taking some of the emphasis off direct face-to-face conversations” (21; p. 2937-8). Two reviews summarized the evidence for the effectiveness of serious games in psychotherapy [16, 18]. The authors concluded that serious games are mostly built on CBT and target a wide range of problems in children and adolescents including attention-deficit hyperactivity disorder (ADHD), autism, anxiety, developmental coordination disorder, impaired social-emotional and communication skills, cyberbullying, impulse regulation, and dyslexia. Both reviews concluded that serious games had a positive impact on the learning of skills and strategies, as well as on alleviating emotional symptoms and stress. In addition, the vast majority of children and adolescents reported high motivation to use serious games, as well as satisfaction with the use of serious games, as part of therapy.

### Serious games used with children and adolescents in treatment for CSA

Surprisingly, there are hardly any serious games that are developed for use in the context of CSA. The games that have been developed focus primarily on prevention [22–24]. One example is Orbit, a CSA prevention computer game targeted at 8- to 10-year-old children [23]. The goal of this adventure game is for the player to do everything they can to help character Sammy that has suffered from sexual abuse. During several mini-games children learn about recognizing CSA, perpetrator tactics, barriers to telling, building a healthy self-concept, and the importance of trusted adults who they can turn to. Researchers currently evaluate the effects of Orbit on children’s knowledge of CSA and how to respond in such situations. In addition, two unpublished master theses describe the development of prototype serious games aimed at helping children to disclose CSA experiences [22, 24]. Pharshy developed a prototype story-telling game in which children can create new stories about their own experiences, or edit existing stories, by using images, drawings, text, and self-created avatars [24]. Parents or caregivers could monitor the child’s stories for possible CSA experiences. Andersson developed a tool for use in a therapy context [22]. This prototype contained different scenes and storylines that therapists could show and play out with children, which could spark conversation about the different (sexual) abuse-related situations children might find themselves in.

The serious game Vil Du?! (Danish for ‘Do you want to [talk about …]?!’) appears to be the first digital game that is being used in a treatment context with child and adolescent CSA victims. Vil Du?! is a non-verbal communication game in which children can show the therapist what happened to them. In the game, which resembles a ‘dress-up game’, both therapist and child operate a self-chosen character, each on their own tablet. The tablets are synchronized to each other, so actions performed on the one screen are also visible on the other, enabling digital interaction between therapist and child. Both players can perform various actions to the other character, by clicking (e.g., undress) or dragging icons (e.g., mouth, hand, penis, buttocks) over the character’s body. Each player can express their boundaries, or pause/stop the game by pressing a Time-Out button. While playing, the therapist can probe the child to talk about its experiences, thoughts, and feelings. The goal of the game was to give children a voice without the need to talk and to put children in charge of creating their own story, normative structure, and values associated to love, sex, and romance.

### Components of cognitive-behavioral therapy for which Vil Du?! might be used

As CBT is most often used in the treatment of CSA victims we will evaluate therapists’ use of Vil Du?! within this context. Although many different forms of CBT can be identified in the literature on CSA treatment (e.g., trauma-focused CBT, game-based CBT, group CBT) several common components can be identified [15, 25–27] for which Vil Du?! might be used. A first important component in most forms of CBT is *psycho-education about sexuality.* Through psycho-education children learn to describe private parts and to identify appropriate and inappropriate touches. This vocabulary can facilitate discussion of children’s CSA experiences later on in the therapy process. In addition, learning vocabulary related to sexuality can also be considered a first step in gradual exposure to the child’s traumatic sexual experiences. Furthermore, psycho-education can enhance future safety of children by teaching them skills to recognize risks and provide them with the language to tell others about future sexual transgressions. Generally, therapists use (anatomical) dolls, pictures, and role play for psycho-education about sexuality [15, 28]. Similarly, the icons of the private parts in Vil Du?! might be used for psycho-education about private parts. In addition, therapists and clients might act out role plays with the characters in Vil Du?! in which the therapist uses the icons to perform (appropriate and inappropriate) actions on the client’s character.

Another component is *trauma-narration and processing* of the sexual abuse experience(s). In order to create and process a narrative of the CSA experiences the therapist invites the child step-by-step to provide increasingly more detailed information about the sexual abuse experiences, as well as associated thoughts, feelings, and behavior. The goal of trauma-narration is to gradually expose children to traumatic memories until children can share their experiences without becoming overwhelmed by negative emotions. The child can create such a narrative orally, in writing, with pictures or with dolls [29]. Similar to doll-play, children might share the story of what happened to them by performing actions on the characters in Vil Du?!.

The trauma-narration process is a gradual process. During the creation of a narrative clients are gradually exposed to trauma reminders. This can help children to master traumatic responses to abuse-related memories, thoughts, and/or feelings. Gradual exposure can for example be done by reading a (fictional/hypothetical) story about CSA or by the therapist reading back parts of the child’s narrative in later sessions. Another way to achieve gradual exposure of children to traumatic memories that might otherwise be avoided is to encourage children to engage in play that realistically depicts their traumatic experiences [30]. Vil Du?! contains explicit icons for the private parts as well as the option to undress the character, which make it possible for children to realistically act out their experiences. In addition, the Time-Out button might be used by client’s to experience control over the exposure to traumatic memories during narrative development sessions [31].

A third component that focuses on enhancing future safety of children is *teaching self-protection skills*. In this context, role plays and hypothetical sexual transgressive situations are often used with the child to practice with saying no, with communicating personal boundaries, and with recognizing intuitive feelings and thoughts that signal imminent danger [15, 31]. The perceived realism of role play is an important predictor of the utility of role plays in interventions [32]. Compared to regular role play, virtual sexual role plays were perceived as more realistic [33] and more effective in learning self-protection skills (i.e., assertive refusal) in threatening sexual situations [34]. In addition, role playing with avatars in a virtual environment (i.e., Second Life) was associated with improvements in psychiatric skills (e.g., management of emotions), expression of less anxiety and more enjoyment than role-playing face to face [35]. Vil Du?! contains male and female characters of different ages which could be used for virtual role plays between therapist and client. The Time-Out button in Vil Du?! might be relevant for the client to express personal boundaries during these role plays.

### Current study

Although Vil Du?! might be a valuable tool for therapy, a practice manual is not yet developed. A practice manual is essential to foster the adoption of this new tool in clinical practice and to train therapists in the use of this new tool [36, 37]. When a practice manual is developed in a research context and is subsequently introduced to practice this might lead to resistance and criticism by clinicians [36]. Amongst others, the so-called research-to-practice-gap may originate from a lack of applicability of a manual or tool to the diverse and complex clinical context [36, 38]. A manual or tool should thus fit within this complex clinical context. Therefore, manuals can be best developed in close collaboration with therapists. This ensures that their needs are met and the gap between research and practice is bridged [39]. In addition, therapists’ evaluations and experiences of working with a new tool or intervention may stimulate the development of a treatment manual as they can discover innovative ways of working with clients [38].

To reduce the research-to-practice gap, we included therapists in the development phase of the user manual by examining whether and how therapists use Vil Du?! with several evidence-based CBT components. This procedure will ensure that the end product is as useful and relevant as possible for clinical practice. Another way to bridge the research-to-practice gap is to conduct qualitative research in the context of psychotherapy [39]. Most importantly, because qualitative methods are well suited to providing an understanding of the individual experience of therapists and clients, which would bring research much closer to the complex and individual context of clinical practice.

This mixed-methods study therefore aims to answer the following research question: How can a serious game designed for helping children open up about sexual abuse experiences be incorporated in psychotherapy for CSA? More specifically, how was Vil Du?! used for specific CBT components (components of CBT treatment models, e.g., psycho-education about sexuality, trauma-narration and processing of CSA experiences, teaching self-protection skills)? With which types of clients Vil Du?! was used, and for which reasons therapists used Vil Du?!

By answering these questions, this study can increase our knowledge on which treatment components for CSA victims are relevant in a serious gaming context. Moreover, the project’s results will be incorporated in a practice manual which sets the stage for further evaluation research on the effectiveness of incorporating serious games, and specifically Vil Du?!, in psychotherapy for young CSA victims with mental health issues. Finally, this research also contributes to the broader literature on the implementation of serious games in psychotherapy for children and adolescents.

## Methods

### Participants

Approximately 20 therapists who were using Vil Du?! at the time of the study were contacted personally, as well as via email, to fill out an online questionnaire every time they used Vil Du?! with a client. We could identify these therapists, because they had received a working license and technical manual for Vil Du?! from the app developers. Therapists completed the online questionnaire for a total of 23 clients with CSA experiences. Mean age of the clients was 11.38 years (*SD* = 3.96, Min = 5, Max = 18). The majority of clients was female (61 %). The total number of therapists participating in the online questionnaire could not be determined because therapists could fill out the questionnaire anonymously. Twelve therapists provided contact information and indicated they were willing to participate in the interview part of the study. Characteristics of the 10 therapists who actually participated in the interviews are presented in Table 1.

**Table 1:** Characteristics of therapists participating in the interview phase of the study

Participant	Age	Years of experience in youth care	Hours per week of therapy with CSA clients or therapy related to sexuality	Work organization^a^
1	42	20	5	1 (Youth care organization providing contextual care for CSA victims)
2	44	15	20	1
3	42	17	32	1
4	32	12	32	2 (Specialized youth care organization focusing on adolescent sexuality)
5	52	30	32	1
6	47	22	16	1
7	28	5	16	1
8	42	17	32	3 (Organization providing specialized family care)
9	62	10	24	1
10	38	17	24	4 (Youth care center for psychotrauma and sexual abuse)

^a^ Participants with the same number in this column work for the same organization

### Design and procedure

We used a mixed-methods triangulation design with quantitative and qualitative data collected and analyzed at approximately the same time. Both types of data were merged and given equal emphasis in the interpretation [40]. Data were collected via online questionnaires (including quantitative and qualitative questions) and semi-structured interviews. Through both methods we derived input from therapists on how and for which CBT components they have been using Vil Du?! for. In the semi-structured interviews we also inquired about other possible uses of Vil Du?! in the context of CBT.

Therapists completed the online questionnaire via Limesurvey. They provided online informed consent for their participation at the beginning of the questionnaire. For the duration of the study (June 2019–June 2020), we invited therapists to complete the questionnaire each time they used Vil Du?! in therapy sessions. Subsequently, a trained graduate student conducted and audio-taped interviews with a selection of the therapists (*n* = 10) who completed the aforementioned online questionnaire. We were able to contact and schedule an interview with 10 out of 12 therapists who indicated their willingness to participate in the interview part of the study.For the interview part of this study, therapists again provided written informed consent before the start of the interview. The interviews took place in a quiet room/office at the work organization of the therapist or online in a quiet home office (due to COVID-19; *n* = 2). The duration of the interviews was on average 45 min. As a preparation for conducting the interviews the graduate student followed university level courses in which semi-structured interviews were practiced. In addition, the first two interviews were conducted under supervision of the principal investigator.

The Ethics Committee of the Faculty of Social and Behavioural Sciences of Utrecht University approved the study.

### Instruments

For both the online questionnaire and the semi-structured interviews, questions and topics were based on common components of trauma-focused CBT that were identified in the literature [31]. When therapists identified a CBT component for which they used Vil Du?! we further inquired about how, and when, in the therapy process they used it. We also asked them about the reasons for using the game, and the types of clients with which they used the game.

#### Online questionnaire

##### Background characteristics

Therapists first had to fill out some background characteristics of the client (i.e., age, gender).

##### CBT components

Participants also indicated, with yes or no, whether they used Vil Du?! for the following therapy components:

1. Psycho-education about sexual abuse (i.e., education about CSA and sexuality, education about personal space and boundaries);
2. Developing and processing a narrative of the CSA experience(s) (i.e., let client tell about CSA experiences, discover details about CSA experience, relive CSA experiences in a safe environment, process CSA experiences in a safe environment); and
3. Teaching self-protection skills (i.e., teaching skills to communicate personal boundaries).

Participants further indicated for these CBT components in how many therapy sessions they used Vil Du?!, when during the course of therapy they used Vil Du?!; and the reason to use Vil Du (i.e., open questions).

#### Semi-structured interviews

The interviews were structured around a topic list, including the following topics that were discussed in more detail than in the online questionnaire:

1. for which treatment components Vil Du?! was used or for which components Vil Du?! seemed a useful tool (i.e., psycho-education, trauma-narration and processing, training self-protection skills);
2. reason(s) for using (or not using) Vil Du?!;
3. how Vil Du?! was introduced to clients;
4. how often and at which moment in the course of therapy Vil Du?! was used.

### Analyses

#### Online questionnaire

SPSS was used to summarize and describe data from the online questionnaires. Descriptive statistics were computed for client age (*M*, *SD*, min, max) and gender (frequency). Frequencies were computed in SPSS to determine the percentage of clients with which Vil Du?! was used for each CBT component. Frequencies were also computed for the type of clients and reasons for which Vil Du?! was used, when in the therapy process Vil Du?! was introduced, and the number of times Vil Du?! was used with each client.

#### Interviews and open questions from online questionnaire

The stepwise guidelines outlined by Zhang and Wildemuth [41] was followed to increase the efficiency, repeatability, and transparency of our qualitative data analysis of the interview data in Nvivo. In step 1, we transcribed answers to all questions literally. Observations during the interview (e.g., sounds, pauses) were not coded, because they were not of interest to the research questions. In step 2, we defined the unit of analysis as themes, i.e., the components of therapy for which and how therapists use Vil Du?!. In step 3, a coding scheme was developed, consisting of CBT components from the literature for which Vil Du?! could be used, the types of clients for which Vil Du?! was used, and therapists’ reasons for using Vil Du?! (the coding scheme is available upon request from the authors). In step 4, the first and second author tested the coding scheme on a sample of text (i.e., 2 pages of text selected from a total of 4 interviews) to discover unclarities in the coding scheme. These were discussed and resolved. In step 5 all text was coded by the first author, while adding new categories to the coding scheme when necessary. For step 6, a set of randomly selected text fragments (20 % of total number of text fragments) was coded both by the first and second author. Differences in coding were discussed until consensus was reached about the category a certain text fragment belonged to. Changes were made to the coding scheme when necessary. The first author recoded the other 80 % of the transcripts on the basis of this changed coding scheme. In step 7, we explored properties and dimensions of the categories as well as relations between the categories in the full range of data. We merged categories that reflected the same content. We also specified category names on the basis of the content that was included in a certain category. Finally, we separated single categories into multiple categories when a category contained different types of information. Answers to the open questions of the online questionnaire were coded under the same categories as the interview data.

## Results

### CBT components in which Vil Du?! was used

Table 2 lists the CBT components for which Vil Du?! was used, ordered from most to least used by therapists.

#### Trauma narration and processing

Both questionnaire and interview data show that Vil Du?! was used by all therapists (*n* = 10) and for the majority of clients (about 60 %) in the context of creating and processing a narrative of traumatic CSA experiences. For example, one therapist mentioned that “Until now I have used it to question children about the details of the abuse, different situations”. Interestingly, 4 therapists also described that the game could be used with parents in the narration process, for example “That you ask the parent, what do you think has happened, show me”. Yet, one therapist thought it would not be that evident to use the game in the narration process with parents.

The trauma narration process differed between clients, 6 therapists described clients who were only able to show their CSA experiences with minimal or no verbal explanation. In contrast, 7 therapists described clients who showed their CSA experiences together with verbal explanation. In addition, during the trauma-narration process therapists gradually exposed their clients to traumatic memories in different ways. For instance, some therapists offered clients to first just show what happened, and invited the client to provide verbal explanations in a second session. Other therapists gradually moved through the disclosure process, by letting the child push the Time-Out button when disclosing became too difficult. This big red button in the middle of the screen pauses the game and takes the payers to a neutral screen. Only in mutual agreement, ‘players’ can go back to the (role)play session. A time out could be followed by relaxation before continuing with the disclosure. Yet other therapists invited clients to monitor and identify emotions while clients showed what happened in Vil Du?!. Asking about emotions further exposes clients to subjective feelings and emotions that are connected to the ‘objective’ events that were shown in the trauma narration phase. A fourth way that was reported was when therapists play out parts of their client’s CSA experiences in Vil Du?! Finally, therapists mentioned using the icons (e.g., of penis, vagina) in Vil Du?! as exposure for assessing the level of trauma.

Additionally, we could identify different types of questions that therapists asked during trauma narration with Vil Du?! Nine therapists mentioned a total of 48 open-ended utterances, such as “What happened then?” or “Where did your uncle look at?”. Six therapists described 17 instances of paraphrasing, such as “You show that the penis goes to the mouth” or “I see that you move the penis along the buttocks”. Five therapists mentioned using 20 option-posing questions (questions that probe the child to choose between two or more options), such as “Is he going inside?” or “Did you undress yourself, or did he do that?”. Finally, 3 therapists recounted a total of 16 suggestive utterances, such as “You did not touch your half-brother, but your half-brother did touch you, right?” or “And then you undressed him/her, right?”. In the context of these suggestive utterances, one therapist specifically mentioned the importance of checking the shared story in a next session and alternating between bogus questions and suggestive questions to avoid getting only affirmative answers from clients.

#### **Psycho-education about sexuality**

For about one third of the clients Vil Du?! was used to provide psycho-education. More specifically, interview data shows that 6 therapists used or would use the game for clients to learn vocabulary related to sexuality and private parts. A therapist mentioned: “Eventually we labelled everything. How do you call this? What does it look like? I call it a vagina, how do you call it?”. However, one therapist mentioned that they would not easily use the game for psychoeducation, but “[…] would rather draw a body and then together explore ‘how is that called?’”. Six therapists used the game for teaching clients about appropriate and inappropriate sexual behaviors. For example, by showing “where you can be touched by whom” or inviting the client to show “where can you be touched? And where not?”.

#### **Teaching self-protection skills**

Vil Du?! was only used with 13 % of the clients for teaching about self-protection skills. Eight therapists thought the game could be used to teach clients to recognize and indicate their sexual boundaries. As described by one therapist:

I think the Vil Du?! app can help. That you put 2 characters in front of each other, a boy and a girl, if it is about indicating boundaries. And what do you do when a boy asks you to do something you do not want to do? How do you react?

However, another therapist was cautious about using the game to teach self-protection skills with specific victims. Especially for victims who already did everything in their power to prevent the CSA experience from happening, teaching self-protection skills might increase feelings of guilt. Only three therapists thought the game could be used to teach clients how to recognize intuitive feelings of danger.

**Table 2:** Questionnaire and interview data about the CBT components in which therapists used Vil Du?!

CBT components in which Vil Du?! was used	Questionnaires	Interviews
	% clients	Therapists (*n*)
*Narration and processing of the CSA experience(s)*		
Q: Let client tell about CSA experiences	61	
Q: Discover details about CSA experience	57	
Q: Process CSA experiences in a safe environment	30	
Q: Relive CSA experiences in a safe environment	13	
I: With clients		10
I: With clients’ parents		4
*Psycho-education about sexuality*		
Q: Education about personal space and boundaries	30	
Q: Education about CSA and sexuality	26	
I: Learning sexuality related vocabulary		6
I: Learning about (in)appropriate sexual behavior		6
*Teaching self-protection skills*	13	
I: Recognizing and indicating boundaries		8
I: Recognizing intuitive feelings of danger		3

Q and I under CBT components represent specific categories or codes that were asked in the questionnaire (Q) or identified in the interviews (I). Questionnaire data represents frequencies computed in SPSS

### The clients and the reasons for which therapists used Vil Du?!

Table 3 lists the types of clients and reasons for which Vil Du?! was used. Reasons to use Vil Du?! were related to specific characteristics of the client (i.e. age, cognitive abilities, psychiatric condition) or characteristics of the therapy process or context (i.e. avoiding by client, experiencing tension by client, discovering details about CSA). Taking these characteristics of clients and the therapy process or context into account, some therapists decided against using Vil Du?!.

In terms of specific characteristics of the clients, we found that most therapists mentioned using the game with adolescents, followed by youth with limited verbal or intellectual abilities, children, parents or family members of CSA victims, and youth with psychiatric conditions. In terms of characteristics of the therapy process or context, the most often mentioned reason to use Vil Du?! was discovering details about the clients CSA experience(s). This was especially the case for clients who found it difficult to talk about these experiences. In this regard, therapists most often described something like: “So, [he was] very defensive, avoidant. It did not become clear what happened in his experience. Therefore, we thought we could use Vil Du?!”. The next often mentioned reason to use Vil Du?! was when clients experienced high levels of tension around the disclosure of CSA experiences. A third reason to use Vil Du?! reported by 3 therapists was that: “The shame and guilt […] were such large barriers to talk to me about what exactly happened. If I would not have had the tablets it would have been much more difficult”. A fourth reason that was brought up by one therapist was related to prevention of CSA or sexual problems: “I think […] if you use it in a preventive way, you have a nice tool to initiate discussion about sexuality and boundaries”. A last reason to use Vil Du?! mentioned by one therapist was that a client liked to play video games.

The most often reported reason to not use or stop using the game was when the game or outlook of disclosing CSA experiences elicits intense negative emotions or signs of re-experiencing in clients. Another reason to not use the game that was mentioned often (4 therapists) is: “If I really have the feeling this child can talk about it super easily, than it does not make sense to use Vil Du?! in my opinion. Then, we can just do it verbally”. Relatedly, when children have strong verbal abilities or high IQ, Vil Du?! appears to be less applicable because these children might find the game childish or unchallenging. Last, using the game with parents of CSA victims might be too confronting, as one therapist told: “parents often want to stay away from the details, and what sense does it make then to use Vil Du?! with parents to show what exactly happened”.

**Table 3:** The type of clients and reasons for which Vil Du?! was used

Type of clients and reasons for which Vil Du?! was used or not	Questionnaires	Interviews
	% clients	Therapists (*n*)
*Type of clients*		
Adolescents (12 years and older)	48	8
Clients with limited verbal or intellectual abilities	4	5
Children (younger than 12 years)	44	5
Parents or family		3
Clients with psychiatric condition (e.g., ADHD)		2
*Reasons to use Vil Du?!*		
Client avoids or is not willing to talk about CSA experiences	17	10
Client experiences high levels of tension around CSA disclosure		6
Client experiences guilt or shame about CSA experiences		3
Prevention		1
Client likes to play video games		1
To discover details about CSA	39	
*Reasons to not use or stop using Vil Du?!*		
Client shows high levels of negative emotions or signs of re-experiencing around CSA disclosure		6
Client could talk or write about events without much emotional difficulty		4
High verbal or intellectual abilities		3
Client finds it difficult to imagine how to use Vil Du?! for showing what happened		1
When parents do not want to know the details about their child’s CSA experiences		1

Percentages under Questionnaires represent frequencies computed in SPSS

### When and how often in the therapy process with a client did therapists use Vil Du?!

There was large variation in the timing of the use of Vil Du?! during the course of therapy. Questionnaire data showed that for some clients (52 % of clients, 4 therapists) Vil Du?! was used in the beginning of the therapy process (i.e., first 5 sessions). Two therapists explained during the interviews that early use of the game is warranted when you need to know the details of CSA as soon as possible to ensure the safety of the client. One therapist described that Vil Du?! makes it possible to talk about CSA experiences earlier in the therapy process than when the game is not used.

For other clients (13 %) the game was used in the middle of the therapy process, or even after 10–20 sessions (22 %). During the interviews, 6 therapists explained that it is important to introduce Vil Du?! not too early in the therapy process, because the therapeutic relationship needs to be built first, especially when clients are highly avoidant. Finally, one therapist reported the use of Vil Du?! like a wildcard, deploying it at the moment “[…] when you think this is not working anymore, this is becoming too difficult”.

Whenever the game is introduced in therapy, it appears important that therapists help clients to get acquainted with the game first. Six therapists mentioned the use of a practice session with clients with Vil Du?! before using it in one of the treatment components. In this practice session they introduced using the icons, choosing the characters, hitting the Time-Out button, talking about the feeling thermometer, entering the session code to start the game. One therapist explained that in the practice session it is important for clients to “first just play around […]. That is also the moment that they can giggle”.

Regarding frequency of use, questionnaire data showed that with the majority of clients (78 %) Vil Du?! was used only once. The rest of the clients used Vil Du?! twice (18 %) or three times (4 %). In the interviews, the majority of therapists thought Vil Du?! could be used multiple times to obtain more detail about the client’s CSA experiences or to discuss different instances of CSA. Other therapists mentioned that Vil Du?! could be used multiple times for different phases or components of therapy. Five therapists thought it was enough to use the game once during the therapy process.

## Discussion

The goal of this study was to examine how a serious game designed for helping children open up about sexual abuse experiences (Vil Du?!) could be incorporated in psychotherapy for young CSA victims with mental health issues. Combining data from questionnaires and semi-structured interviews, we found that Vil Du?! was used for several CBT components in psychotherapy for CSA victims. More specifically, therapists applied the game for narration and processing of the CSA experience(s), psycho-education about sexuality, and teaching self-protection skills. The therapists in this study provided many recommendations for the use of Vil Du?! for these CBT components, which subsequently will be discussed in more detail. However, these recommendations need to be viewed in light of therapists personal avoidance strategies during therapy. It is known that therapists working with traumatized clients often experience symptoms of avoidance themselves [42] and avoid discussing traumatic events because they fear negative emotions in their clients, their parents, or themselves [43].

Vil Du?! was used most often in the context of trauma narration and processing. This is not surprising because the main goal of the game was to make it easier for children to share their CSA experiences. Creating a trauma narrative of CSA experiences is a critical component of trauma-focused CBT [31], but also in other CBT interventions such as writing therapy [44]. Clinical guidelines also state the inclusion of elaboration and processing of the traumatic memories in the treatment of CSA victims with mental health symptoms [12, 13]. Trauma narration might be such an essential component of therapy, because therapists can build on the narrative in several other CBT components such as gradual exposure to the traumatic memories and restructuring maladaptive thoughts in the narrative [31]. There is some previous evidence that trauma narration might be the critical mechanism for producing positive outcomes after CSA, especially with regard to children’s abuse-related fear and general anxiety [45]. Children themselves also often mentioned that talking, drawing or writing about the sexual abuse specifically was the most helpful part of therapy [25, 45]. Vil Du?! may contribute to trauma narration by offering an alternative communication tool. This tool may supplement the talking, writing and drawing with playful interactions that are closely related to doll play.

Therapists in this study shaped the trauma-narration process in a gradual way and they used Vil Du?! for this gradual process in different ways. These differences could be related to the phase in the trauma-narration process the therapists were in with certain clients. Trauma-narration activities often are organized in a hierarchical way to ensure that clients do not become overwhelmed by negative emotions related to the traumatic memories [15, 31]. We identified the following steps in the hierarchy of increasingly explicit trauma-narration activities that are possible with Vil Du?!:

1. Using the icons (e.g., for penis, vagina, buttocks) in Vil Du?! as exposure to traumatic memories;
2. Passive disclosure by showing what happened, followed by more active disclosure by verbally explaining what clients show in Vil Du?!;
3. Gradual active disclosure of different aspects of CSA experiences, by letting the client push the Time-Out button when disclosing becomes too difficult, followed by relaxation and continuation of disclosure (not continuing the disclosure process might reinforce avoidance in clients);
4. Disclosure of emotions when the client shows what happened in Vil Du?!;
5. Therapists play out parts of their client’s CSA experiences in Vil Du?!.

This hierarchy of activities is similar to the exposure hierarchy in game-based CBT [15]. Even though Vil Du?! might be used as a tool in several trauma-narration activities (i.e., to work toward gradual exposure to traumatic memories), it cannot completely replace the trauma narrative. Further processing of the narrative, via talking, writing, or drawing, might be necessary to fully expose clients to all aspects (i.e., thoughts, feelings, behaviors) of the traumatic memories [31].

During trauma narration with Vil Du?! therapists asked different types of questions to their clients. It was important to examine these questions because there is evidence that questioning children about CSA experiences with the use of dolls or body drawings can increase the risk of incorrect reports, especially when suggestive questions are asked [46, 47]. Trauma narration with Vil Du?! resembles questioning children about CSA experiences with dolls. We found that therapists mostly used open-ended utterances to obtain details about their clients’ CSA experiences. This may mitigate the risk of incorrect reports because open-ended prompts are seen as the golden standard in interview techniques for accurate disclosure of CSA experiences [48]. If crucial details about events are still missing, option-posing questions can be asked [49], which is something the therapists in our study did as well. Therapists in our study also relied on more suggestive utterances. Suggestive utterances may help clients to find words and to continue the disclosure process. Vil Du?! offers clients an alternative to talking by enabling them to show what happened. According to the therapists in this study many clients indeed provided hardly any verbal explanation of what they showed in Vil Du?!. This might relate to one of the most often mentioned reasons for using Vil Du?!, i.e., that children do not want to talk about CSA experiences. In the non-verbal communication context that Vil Du?! offers it seems logical that therapists revert to suggestive utterances. Yet, suggestive utterances are known to be related to inaccurate recollections of events (for a review, see [48]) and are therefore strongly discouraged in validated interview protocols for CSA [49]. Therefore, the amount of suggestive questions used by therapists was somewhat alarming. It is advisable, as one therapist mentioned, to check the shared story with the client in a next session, or to alternate between asking bogus questions (i.e., to which the client has to answer no to) and suggestive questions.

It is important to point out that even though many clients provided hardly any verbal explanation of what they showed in Vil Du?!, the majority of therapists also recounted situations in which clients were able to verbally narrate what they showed in Vil Du?! It could be that Vil Du?! might have functioned as a ‘third party in the room’, making the trauma-narration process less difficult for clients because there was no need for direct face-to-face conversations [21]. Therefore, clients may have been more at ease to discuss their experiences. This hypothesis remains to be tested in future research.

Some therapists also described using Vil Du?! for trauma narration with the (non-abusing) parents of their clients. They applied the game to obtain the parents’ perspective on what happened. This fits with the parent sessions included in trauma-focused CBT in which parents discuss the details they already know about their child’s CSA experiences with the therapist [31] and with clinical recommendations to involve non-offending parents in the therapy process [13]. Creating a shared understanding through a shared story can facilitate clients’ recovery after CSA. It is therefore important for parents to process their own narrative in order to avoid negative reactions to their child’s narrative [50]. However, according to some of the therapists in our study, using Vil Du?! with parents is not advisable when parents do not want to know the details about their child’s CSA experiences, because this might be too disturbing.

Vil Du?! was used less for psycho-educating clients about sexuality-related vocabulary and (in)appropriate sexual behaviors and for teaching clients self-protection skills related to indicating boundaries and recognizing intuitive feelings of danger. This was not expected because the game-based and explicit visual nature of Vil Du?! appeared very suitable to teach children about sexuality. In addition, the interactive nature of the game, with characters of different ages that could be used for role plays, appeared to enable clients to practice self-protection skills. An explanation for the lower than expected use of Vil Du?! for these CBT components could be that most therapists mentioned using Vil Du?! in the context of CBT writing therapy [44]. This treatment does not include CSA-specific CBT components such as psycho-education about sexuality and teaching of self-protection skills. Incorporation of Vil Du?! in the CBT components of psycho-education and teaching self-protection skills might still be valuable because (non-digital) game-based CBT, which includes both psycho-education and practicing with self-protection, has been found to reduce a wide range of negative outcomes following CSA [51]. Moreover, according to clinical guidelines treatment for CSA victims with mental health issues should focus on psycho-education as well as promoting safety [12, 13]. In addition, practicing self-protection skills in a virtual context such as Vil Du?! might enhance the enjoyment and effectiveness of role plays [34, 35]. Yet, as recommended by one therapist, the use of Vil Du?! might not be warranted for teaching self-protection skills to victims who already did everything in their power to stop the sexual abuse, in order to prevent increased feelings of guilt.

The reasons for which therapists used Vil Du?! for the most part refer to their clients experiencing barriers toward disclosure. Barriers included a lack of verbal abilities, or feelings of shame, guilt, avoidance, and tension. Talking about CSA experiences is known to be difficult for these reasons (amongst others) [52]. It also makes sense that therapists mentioned not using Vil Du?! when clients do not experience these barriers, because such clients might see the game as less useful. In addition, the game could be boring or childish for clients with high intellectual or verbal abilities. Therefore, according to therapists the game should preferably not be used with such clients.

Because many clients experience barriers to disclose CSA, most therapists mentioned the importance of not introducing Vil Du?! too early in the therapy process. According to therapists, a good therapeutic alliance needs to be built first. Building such an alliance takes time. Time to build a therapeutic alliance is considered rather important since length of time in therapy and strength of the therapeutic alliance predict overall disclosure in therapy [53]. Not only is it important for therapists to build a trusting relationship with their client, clients also need to trust the interaction with VilDu?!. In order to create a sense of self-efficacy in relation to the game, therapists mentioned the use of a practice session. In a practice session clients can play around with the icons and characters, or have fun with the game. Such play in which the child is in control is known to reduce tension and stress [54], and may strengthen the alliance between therapist, client, and game.

Regarding frequency of use, most therapists used Vil Du?! only once in the therapy process, even though they thought it *could* be used multiple times or for multiple CBT components. It is possible that therapists might be unaware of the broad employability of Vil Du?! or they were unsure how to use Vil Du?! for different components of therapy. A user manual which describes in detail how other therapists have used Vil Du?! for different treatment components could help to overcome these barriers. That therapists mentioned that the game *could* be used multiple times, even though they themselves mostly used it once in each therapy process, could also be an indication of social desirable responding during the interviews.

### Limitations of the study and directions for future research

An important limitation of this study is that we focused mainly on the therapists’ perspective in the use of Vil Du?!. Yet, clients themselves might think differently about how the game could be used in therapy. Future research could include the clients’ perspective by asking clients to fill out short, age-appropriate evaluation questionnaires following the use of a serious game in psychotherapy. A second limitation is that we were not able to include therapists who represented a wide range in terms of the therapy type they used and the organization they worked for. The majority of participating therapists were working for the same organization, and thus with the same treatment protocols. This may have limited the ways in which therapists used Vil Du?! Relatedly, the background information that we collected on therapists and their clients was rather limited. It could be that Vil Du?! was used very differently for patients with different psychiatric conditions or depending on the experience therapists had with using Vil Du?! These questions might be interesting to examine in future studies.

Another important future direction for research on Vil Du?! is to conduct experimental or longitudinal research to examine the effectivity of incorporating Vil Du?! in CSA treatment. These studies could also examine the specific working elements underlying the effects Vil Du?! might have on the therapy process. However, in order to take this next step in evaluating the effects of Vil Du?! the findings of the present study will be incorporated into a user manual for Vil Du?!. With this manual, therapists can use the game in a more systematic way (access to the manual and Vil Du?! can be arranged through the authors). Next steps in the broader implementation of Vil Du?! could be to explore the useability of the game for prevention purposes (e.g., psycho-education about sexuality and appropriate and inappropriate touching in schools) and early detection of sexual abuse (e.g., use during consultations with children in child welfare clinics). In addition, text in the game environment and the manual need to be translated in different languages.

### Conclusion﻿s

To conclude, this study provides new information about the incorporation of serious games, and specifically a game designed for helping children open up about CSA experiences (i.e., Vil Du?!), in psychotherapy for young CSA victims with mental health problems. Therapists acknowledge the usefulness of Vil Du?! for several CBT components, but mostly for trauma narration and processing, and psycho-education about sexuality. For the trauma narration component, therapists mostly used open-ended utterances to obtain details about children’s CSA experiences, which is considered the golden standard in interview techniques for accurate disclosure of CSA experiences. Vil Du?! might be most useful for clients who experience barriers toward disclosing their CSA experiences. Barriers include, but are not limited to, a lack of verbal abilities, or feelings of shame, guilt, avoidance, and tension. The recommendations from this study have been incorporated in a manual. This manual is an important step towards more systematic and broader implementation of Vil Du?! in the therapy of child and adolescent victims of CSA. Important next steps are to examine the working elements of Vil Du?! and to test whether implementing Vil Du?! in therapy is effective in reducing the negative mental health consequences of CSA, such as PTSD and sexualized behaviors, for children and adolescents.

## Data Availability

Transcripts of interviews will not be shared because study participants did not give their approval in the informed consent.

## Abbreviations

CSA: Child sexual abuse
PTSD: Post-traumatic stress disorder
CBT: Cognitive behavior therapy
ADHD: Attention-deficit hyperactivity disorder
